# 

**Published:** 1847-10

**Authors:** 


### CONTENTS.

PART I.—ORIGINAL COMMUNICATIONS.

ART. 1. *Tannate of Quinia.* By John McLean, M. D., Prof., &c.

ART. 2. *Etherization applied to Midwifery.* By J. E. McGirr, M. D.

ART. 3. *Strychnine in Fevers.* By A. W. Benton, M. D., Sterling Ill.

ART. 4. *Surgery at Buena Vista.* By W. B. Herrick, M. D., Professor of Anatomy in Rush Medical College, and late Surgeon 1st. Regiment Illinois Volunteers.

ART. 5. *A Case of Glossitis—Maltreatment, nearly resulting in death.* By T. P. Bichnell, M. D.

ART. 6. *Case of Accumulation of Fluid in the Ureter and Pelvis of the Kidney, upon the right side, of long standing; followed by retention and suppression of urine, acute inflammation of the bladder and left kidney, terminating in suppuration and death.* By W. B. Herrick, M. D., Professor of Anatomy in Rush Medical College.

ART. 7. *Physical Condition of the Aborigines—with an account of their practice of medicine,* By Edwin G. Meek, M. D., of Choctaw Agency.

PART II.—REVIEWS.

ART. 8. *A Practical Treatise on Inflammation, Ulceration and Induration of the Ne k of the Uterus—with Remarks on the value of Leucorrhœa and Prolapsus Uteri, as symptoms of uterine disease.* By James Henry Bennett, M. D., Licentiate of the Royal College of Physicians, etc., etc. Philadelphia: Lea & Blanchard.

ART. 9. *A Treatise on the Practice of Medicine.* By Geo. B. Wood, M. D., Professor of Materia Medica and Pharmacy in the Uuiversity of Pennsylvania, etc., etc.

PART III.—BIBLIOGRAPHICAL NOTICES.

ART. 10. *Elementary Chemistry, Theoretical and Practical.* By Geo. Fownes, Ph. D., Chemical Lecturer in the Middlesex Hospital School, etc. Edited with Additions by Robert Bridges, M. D., etc.

ART. 11. *An Analysis of Physiology; being a condensed view of its more important facts and doctrines.* Designed especially for the use of students. By John J. Reese, M. D., etc., etc.

PART IV.—SELECTIONS.

1. Adulteration of Medicines.
2. Alum in Pertusis.
3. Apothecaries.
4. Influence of terrestial and atmospheric Electricity upon the system.
5. Hereditary Transmission of Insanity.
6. New Method for the Union of Wounds.
7. Burns treated with Ammonia.
8. Influence of Coffee upon the Sulphate of Quinine.
9. Means of ascertaining if Alcohol be perfectly pure.
10. Method of detecting the presence of Cotton in Linen.
11. Shetches and Illustrations of Medical Quackery.
12. On Etherization.

13 & 14. Croup.

15. Gastric Origin of Croup.
16. Medical Heroism
17. The Fate of the Physician.
18. A mode of Resusitating Patients after inhaling the Vapor of Ether.
19. On the Use of the Muriate of Morphine in Toothache, etc., etc.
20. Rape perpetrated on a female while under the influence of ether.
21. Anecdote of an insane clergyman before the American Revolution.
22. University of Pennsylvania.

22\. Medical College of Ohio.

PART V.—EDITORIAL.

ART. 1 The System of "Concours."

ART. 2. On the inhalation ot Sulphuric Ether.

